# Evaluating bowel preparation tolerability in IBD: a phase 3 *post-hoc* comparison of mannitol and PEG-ASC

**DOI:** 10.3389/fgstr.2025.1630479

**Published:** 2025-08-25

**Authors:** Gian Eugenio Tontini, Alessandro Rimondi, Flavio Caprioli, Giovanni Aldinio, Luca Pastorelli, Franco Radaelli, Cristiano Spada, Maurizio Vecchi

**Affiliations:** ^1^ Department of Pathophysiology and Organ Transplantation, University of Milan, Milan, Italy; ^2^ Gastroenterology and Endoscopy Unit, Foundation IRCCS Ca’ Granda Ospedale Maggiore Policlinico, Milan, Italy; ^3^ Royal Free Unit for Endoscopy, Royal Free London NHS Foundation Trust, London, United Kingdom; ^4^ Department of Health Sciences, University of Milan, Milan, Italy; ^5^ Gastroenterology Unit, Valduce Hospital, Como, Italy; ^6^ Digestive Endoscopy Unit, Fondazione Policlinico Universitario Agostino Gemelli IRCCS, Rome, Italy; ^7^ Fondazione Poliambulanza Istituto Ospedaliero, Brescia, Italy

**Keywords:** bowel preparation, inflammatory bowel disease, colonoscopy, oral mannitol, polyethylene glycol, patient tolerability, ulcerative colitis, Crohn’s disease

## Abstract

Bowel preparation is a significant challenge for patients undergoing colonoscopy, especially in Inflammatory Bowel Disease (IBD) patients. Oral mannitol (OM), an ultra-low-volume, single-dose osmotic laxative with a sweet taste, could improve patient tolerability. We performed a *post-hoc* analysis of the phase 3, multicentre, randomized, endoscopist-blinded SATISFACTION trial to compare same-day OM versus standard split-dose 2L polyethylene glycol plus ascorbate (PEG-ASC) for bowel preparation in IBD patients. Fifty-five IBD patients (24 OM, 31 PEG-ASC) were assessed for bowel cleansing efficacy, endoscopic outcomes, safety, and patient satisfaction. OM demonstrated superior tolerability, including ease of use (88% *vs*. 71%), taste satisfaction (75% *vs*. 6%), and willingness to reuse (96% *vs*. 71%). OM also reduced intake duration (32 *vs*. 107 minutes) and time to evacuation (57 *vs*. 91 minutes), with comparable efficacy, cleansing quality, and safety. In conclusion, same-day OM preparation enhances satisfaction and adherence in IBD patients, offering a safe, effective alternative to standard protocols.

## 1 Introduction

Colonoscopy plays a pivotal role in the diagnosis and monitoring of inflammatory bowel diseases (IBD). The diagnosis of both ulcerative colitis (UC) and Crohn’s disease (CD) requires lifelong exposure to colonoscopy to assess disease activity and extent, as well as for colorectal cancer surveillance (1).

Although endoscopy-related anxiety is generally managed and overcome by IBD patients, in some cases, adherence to colonoscopy remains very poor due to the discomfort caused by bowel cleansing. Current bowel preparation strategies based on low volumes of polyethylene glycol (PEG) and a split regimen guarantee high efficacy and a favourable safety profile, but suboptimal patient-reported outcomes are mainly related to the solution’s salty taste and the regimen’s complexity (2–4).

A large multicentre phase 3 trial has recently evaluated both the safety and the efficacy of a novel ultra-low-volume, mannitol-based preparation (oral mannitol, OM) administered 4 hours before colonoscopy. Remarkably, this novel preparation met the ESGE quality standard efficacy threshold leading to adequate bowel cleansing in over 90% of patients and showed excellent performance in terms of patients’ adherence, tolerability and ease of use (5).

We conducted a *post-hoc* analysis of the SATISFACTION phase III study to explore whether, in IBD patients, a single dose of OM administered 4 hours before colonoscopy might represent a valid alternative to the standard split-dose 2L polyethylene glycol plus ascorbate (PEG-ASC) preparation in terms of bowel cleansing efficacy, safety and patient acceptance.

## 2 Materials and methods

This is a *post hoc* analysis derived from the SATISFACTION study, a phase III, international, multicentre, randomized, parallel-group, endoscopist-blinded trial conducted across 30 centres between March 2021 and July 2021 (5). The trial protocol was registered with ClinicalTrials.gov (https://clinicaltrials.gov/ct2/show/NCT04759885) and EudraCT (eudract_number:2019-002856-18) and was finally approved by each local Ethics Committee.

**Figure 1:**
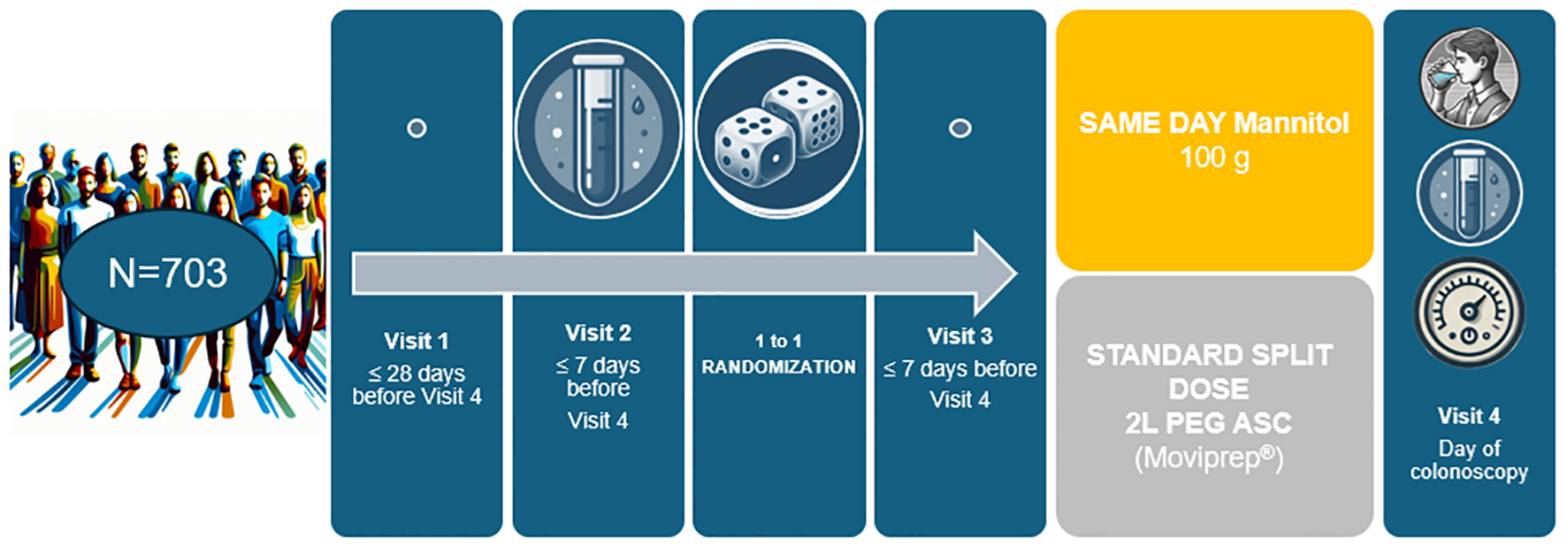
The SATISFACTION study: Patients were randomly assigned in a 1:1 ratio to one of two treatment groups: 1) 100 g of mannitol powder dissolved in 750 mL of water, to be consumed within 30 minutes on the day of the colonoscopy. This solution had to be completed at least 4 hours prior to the procedure. Following this, patients were allowed to drink an additional 1 L of clear liquids to prevent dehydration; 2) 2L of Polyethylene Glycol with Ascorbate (PEG-ASC) in a split-dose regimen. The first litre was consumed over 1–2 hours the evening before the colonoscopy, and the second litre on the morning of the procedure, ensuring consumption was completed at least 4 hours prior to the colonoscopy. After each dose, patients were required to drink 500 mL of clear liquids. Additionally, they were encouraged to consume at least 1 L of extra clear liquids to mitigate thirst and prevent dehydration. For both arms, blood samples for standard haematology, clinical chemistry and electrolyte levels were taken ≤7 bays before and on the colonoscopy day and measurements of intestinal gas concentrations (H_2_, CH_4_ and O_2_), using a multi-gas detector (Dräger X-am 8000), were taken on the colonoscopy day. Experienced endoscopists performing study colonoscopies were blinded to the treatment allocation.

The original phase III study was designed based on the basis of previous dose-finding (6) and pharmacokinetics analyses (7) to demonstrate the non-inferiority of a low-volume (100gr/750mL), single-dose OM administered 4 hours before colonoscopy compared to a split-dose 2 L PEG-ASC preparation in terms of bowel cleansing efficacy and its superiority in terms of patient acceptance. Patients receiving OM in the phase II and in the phase III studies and those receiving PEG-ASC in the phase III study underwent measurements of intestinal O_2_, H_2_ and CH_4_ levels at different colonic segments to demonstrate the absence of potentially critical gas concentrations regardless of bowel preparation regimens and solutions (8) (**Figure 1**).

For the current analysis, data regarding all IBD patients enrolled in the SATISFACTION study were evaluated. The randomized design of the original study was maintained for the comparison of treatment groups and no additional interventions were performed. This study was conducted in accordance with the CONSORT guidelines for randomized trials (9).

Inclusion criteria required patients to be over 18 years of age, able to provide signed informed consent, and scheduled for an elective colonoscopy (screening, surveillance, or diagnostic) to be performed according to ESGE guidelines (4).

Exclusion criteria included pregnancy or breastfeeding; severe renal impairment (estimated glomerular filtration rate [eGFR] <30 mL/min/1.73 m²); severe heart failure (New York Heart Association [NYHA] Class III–IV); severe anaemia (Hb <8 g/dL); clinically active inflammatory bowel disease; advanced chronic liver disease (Child-Pugh class B or C); significant electrolyte disturbances; recent (<6 months) symptomatic acute ischemic heart disease; history of major gastrointestinal surgery; and current use of laxatives or medications affecting colonic motility.

The primary endpoint was the proportion of patients with adequate bowel cleansing, defined as a Boston Bowel Preparation Scale (BBPS) total score ≥6, with a score for each of the three colon segments (right; transverse, including flexures; left, including the sigmoid and rectum) ≥2. The secondary endpoints were adenoma detection rate, caecal intubation rate, time to evacuation, time to complete treatment intake, rate of complete study drug intake, taste (numeric rating scale [NRS]: 0 = terrible to 10 = very good), ease of use (NRS: 0 = very difficult to 10 = very easy), willingness to repeat the preparation with the same product (yes/no) and treatment-emergent adverse events.

Continuous numerical variables were tested for normality and analysed using Student’s T test. Given the small number of patients in this *post-hoc* analysis, the categorical variables were tested for statistically significant differences using Fisher’s exact test. All the statistical analyses were performed with R version 4.4.0 (R Core Team (2024),; *R: A Language and Environment for Statistical Computing;* R Foundation for Statistical Computing, Vienna, Austria; https://www.R-project.org/ ).

## 3 Results

**Figure 2:**
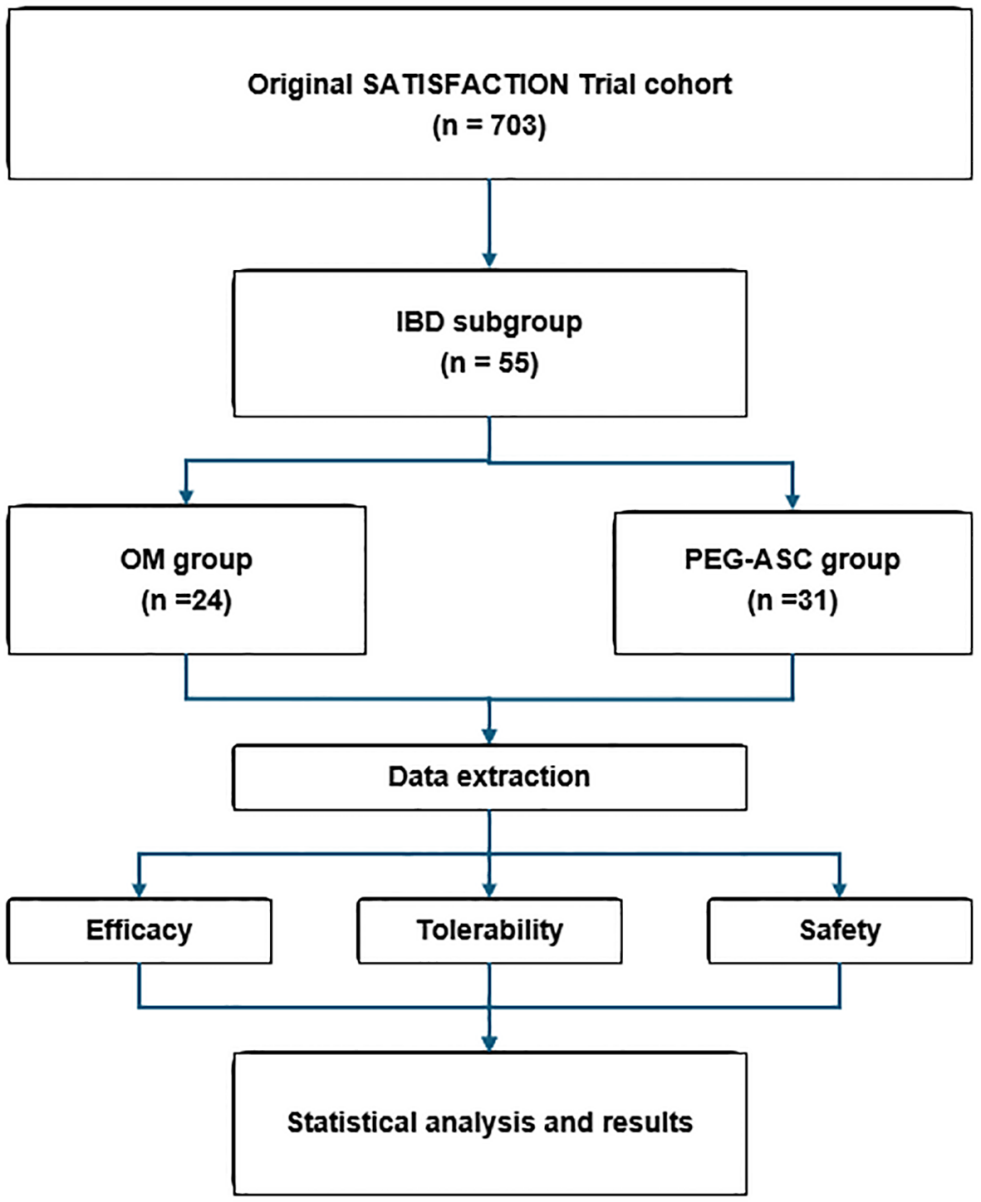
Flowchart of the SATISFACTION trial *post-hoc* analysis: All inflammatory bowel disease (IBD) patients in the original RCT were included. The resulting IBD subgroup was divided based on prior randomization to oral mannitol (OM) or polyethylene glycol with ascorbate (PEG-ASC). The *post-hoc* analysis included data extraction of efficacy, safety, and tolerability endpoints, followed by statistical analysis.

A total of 55 participants diagnosed with IBD were included in the analysis, with 24 assigned to the OM group and 31 to the 2L PEG group (**Figure 2**). The mean age in the OM group was 48.9 years (SD = 14.9), compared to 44.5 years (SD = 14.2) in the 2L PEG group. Sex distribution was also similar, with females representing 45.8% of the OM group and 45.1% of the 2L PEG group. Almost all participants were non-Hispanic and non-Latino, with the exception of a single participant in the OM group.

Disease distribution was comparable across the two cohorts: 18 patients in the OM group and 23 in the 2L PEG group were diagnosed with colitis (OM: 17 with UC, 1 with microscopic colitis; 2L PEG: 21 with ulcerative colitis, 2 with microscopic colitis), while 6 and 8 patients were diagnosed with CD in the OM and 2L PEG groups, respectively (p = 1). For more information, refer to **Table 1**.

**Table 1:** Study population.

Characteristics	OM (n = 24)	2L PEG (n = 31)	P value
Age, years, mean (SD)	48.9 (14.9)	44.5 (14.2)	0.27
Female, n (%)	11 (45.8)	13 (41.9)	0.79
Disease, n (%)			
Ulcerative colitis Microscopic colitis Crohn’s disease	17 (70.8) 1 (4.2) 6 (25.0)	21 (67.7) 2 (6.5) 8 (25.8)	1
Endoscopic severity			
Ulcerative colitis [according to Mayo endoscopic subscore (10)], n (%)			
Remission Mild Moderate Severe	8 (47.1) 5 (29.4) 3 (17.6) 1 (5.9)	16 (76.2) 3 (14.3) 2 (9.5) 0 (0.0)	0.29
Crohn’s disease [according to SES-CD (11)], n (%)			
Inactive (0-2) Mild (3-6) Moderate (7-15) Severe (≥16)	3 (50.0) 2 (33.3) 1 (16.7) 0 (0.0)	4 (50.0) 1 (12.5) 3 (37.5) 0 (0.0)	0.79
Disease extent [according to Montreal Classification (12)], n (%)			
Ulcerative colitis			
Proctitis Left-sided Extensive	4 (23.5) 6 (35.3) 7 (41.2)	3 (14.3) 5 (23.8) 13 (61.9)	0.50
Crohn’s disease			
Terminal ileum Colon Ileocolon	5 (83.3) 1 (16.7) 0 (0.0)	0 (0.0) 3 (37.5) 5 (62.5)	0.003
Indication, n (%)			
Diagnosis Monitoring Surveillance	1 (4.2) 17 (70.8) 6 (25.0)	1 (3.2) 14 (45.2) 16 (51.6)	0.07
Adenoma, n (%)	0 (0)	1 (3.2)	1
Serrated sessile lesion, n (%)	0	0	1
Crohn’s disease with small bowel stenosis	2 (33.3)	2 (25.0)	1

OM, oral mannitol; PEG, polyethylene glycol; SD, standard deviation; SES-CD, simplified endoscopic activity score for Crohn’s disease.

All patients followed a low-fibre diet for at least three days prior to the examination.

Adequate bowel cleansing (BBPS ≥2 in each large bowel segment) was achieved in 95.8% and 96.8% of patients receiving OM and 2L PEG-ASC, respectively (OR 0.77, 95% CI 0.01-62.7). No statistically significant differences were found in terms of other colonoscopy performance measures between the two cohorts (**Table 2**).

**Table 2:** Performance measures and patient reported outcomes in the oral mannitol (OM) and 2L polyethylene glycol (PEG) cohorts.

Performance measure	OM (n = 24)	2L PEG (n = 31)	OR (95%CI)	P value
Caecal intubation, n (%)	24 (100)	31 (100)	0 (0-Inf)	1
Right colon BBPS ≥ 2, n (%)	23 (95.8)	30 (96.8)	0.77 (0.01 – 62.7)	1
Transverse colon BBPS ≥ 2, n (%)	24 (100)	31 (100)	0 (0-Inf)	1
Left colon BBPS ≥ 2, n (%)	24 (100)	31 (100)	0 (0-Inf)	1
Adequate bowel cleansing, n (%)	23 (95.8)	30 (96.8)	0.77 (0.01 – 62.7)	1
BBPS,mean (SD)	8.3 (1.22)	8.3 (1.22)	–	1
Patient reported outcome				
Taste (NRS > 8), n (%)	18 (75.0)	2 (6.4)	41.8 (7.34 - 461.06)	< 0.001
Taste (NRS > 6), n (%)	21 (87.5)	14 (45.2)	8.15 (1.86 – 51.57)	0.001
Willingness to reuse preparation, n (%)	23 (95.8)	22 (71.0)	9.09 (1.1 – 428.98)	0.03
Easy to use (NRS > 8), n (%)	21 (87.5)	22 (71.0)	2.81 (0.59 – 16.36)	0.19
Easy to use (NRS > 6), n (%)	23 (95.8)	26 (83.9)	4.32 (0.44 – 217.9)	0.21
Completeness of bowel preparation, n (%)	24 (100)	29 (93.5)	Inf (0.14-Inf)	0.49

OR, odds ratio; CI, confidence interval; SD, standard deviation; Inf, infinite; BBPS, Boston Bowel Preparation Scale; NRS, numeric rating scale.

Mean duration of study treatment intake was 31.6 minutes (SD = 12.81) in the OM group and 107.2 minutes (SD = 37.16) in the 2L PEG group (p < 0.001).

Mean time between the study treatment intake start and the occurrence of the first evacuation was 56.6 minutes (SD = 34.57) in the OM group and 90.7 minutes (SD = 46.51) in the 2L PEG group (p < 0.001).

A significant difference between the two cohorts was found in the patient reported outcomes: the OM group outscored the 2L PEG in terms of *taste* (p= 0.001) and *willingness to reuse* the same preparation (p = 0.03).

All patients who received OM had *full adherence* to the bowel prep regimen, completing the whole bowel prep, whereas 2 patients in the 2L PEG group managed to take only partial bowel prep (p = 0.49).

No statistically significant difference in the *ease of use* was found between the two cohorts (**Table 2**).

A single adenoma was found in the group that received 2L PEG (p = 1): a non-pedunculated polyp measuring less than 5 mm located in the transverse colon, histologically classified as a tubular adenoma with low-grade dysplasia. No serrated sessile lesions were identified.

Treatment-emergent adverse events occurred rarely in both groups and mostly related to nausea and vomiting (4 OM, 1 PEG; p = 0.15). One patient in the OM group prematurely discontinued the preparation due to vomiting.

Biopsies were usually taken in each colonic segment according to local protocols and ECCO guidelines (1). The histopathology reports were consistent with endoscopic findings and with IBD diagnosis. No inflammatory or architectural tissue change was described as suggestive for a drug-induced aetiology potentially referable to an effect of the laxative therapy.

## 4 Discussion

This *post-hoc* analysis addressed whether a single-dose, ultra-low-volume OM regimen is a safe, effective, and more tolerable alternative to the standard split-dose 2L PEG-ASC preparation for bowel cleansing in patients with IBD. By directly comparing these regimens in an IBD population from the SATISFACTION trial, we aimed to determine if OM could improve patient satisfaction and adherence while maintaining efficacy and safety, in line with the ideal bowel preparation for IBD patients, which should be effective, safe, easily self‐administered and well-tolerated (13). The two groups were balanced for age, sex, and distribution of disease (ulcerative colitis and Crohn’s disease), ensuring comparability of the results.

Both regimens achieved high rates of adequate bowel cleansing according to BBPS (OM 95.8%, PEG-ASC 96.8%), with no significant differences. No significant differences were observed in other endoscopic performance measures between the two cohorts, including caecal intubation rate and adenoma detection rate. Specifically, a single adenoma was found in the PEG-ASC group, with no statistically significant difference. This likely reflects the low baseline risk of neoplasia and the relatively young mean age of the enrolled population.

This finding confirms previous literature showing that low-volume preparations are effective in IBD patients, and demonstrates that even non-PEG, single-dose regimens can ensure adequate cleansing (>90%) and meet ESGE standards (4, 14).

Achieving an adequate bowel cleansing (i.e., segmental Boston bowel preparation scale ≥ 2) represents the minimum standard for appropriate endoscopic assessment and minimizes the need for repeat procedures in a short time frame. Optimal mucosal visualization (e.g., BBPS ≥8) is essential for precise assessment of disease activity and, to a greater extent, for early dysplasia detection in surveillance follow-up together with advanced endoscopic chromoendoscopy techniques (1, 15, 16).

The split-dose administration resulted in better visualization scores and patients’ acceptance in many IBD studies and it is currently recommended by international guidelines (1).

To date, data for very low-volume (< 2 L) bowel preparations are scant and limited to PEG-based regimens with adjuvants (17). Moreover, non-PEG sulphate-based options demonstrated efficacy and tolerability outcomes comparable to low-volume PEG-based preparations in patients with IBD (18), but resulted in a 10-fold increase in macroscopic mucosal inflammatory changes (3.3-3.5% absolute risk) (4), which hinders their routine use in this specific setting. This undesirable event is usually associated with the use of either contact laxatives or osmotic laxatives with marked hyperosmolarity. In contrast, no mucosal lesion has ever been reported with the use of sugar-based low-volume preparations, which are characterized by mild hyperosmolarity (5–7). Notably, the osmolarity of 100 g mannitol in 750 ml of water is equal to 0.787 Osm/Kg, corresponding to an intermediate level between the osmolarities of standard PEG solutions (up to 270 Osm/Kg for 4L PEG and 400 Osm/Kg for 2L PEG) and the osmolarity of non-PEG sulphate solution (up to 1070 Osm/Kg) or 1L PEG-ASC (up to 1174 and 1472 Osm/Kg for preparation A and preparation B, respectively).

The OM group demonstrated superior tolerability, including significantly better ratings for taste and a higher willingness to reuse the same preparation in the future. Consistently, all patients who received the same day regimen with OM completed the full bowel preparation, whereas two patients in the PEG-ASC group managed to take only a partial preparation despite being administered in a split regimen.

This finding clearly reflects the strong preference for the mannitol sweet flavour rather than for the PEG salty taste, as well as the practicality of the same-day regimen that reduces both the duration of intestinal preparation and patients’ limitations in everyday life.

Mean duration of study treatment intake was substantially lower in the OM group (32 minutes *vs*. 107 minutes), as was the mean time from preparation start to first evacuation (57 *vs*. 91 minutes).

These features are likely to reduce perceived discomfort and may improve the overall patient experience, especially for those who require frequent endoscopic examinations.

Milder expected drug-related adverse events such as nausea, vomiting and abdominal pain were substantially uncommon and were observed to a greater extent, although not significantly, in the group subjected to same-day preparation with OM. Overall, both regimens appeared safe and well tolerated, with no serious adverse events.

We acknowledge that the results of our *post-hoc* analysis on a relatively small sample of patients cannot be generalized. Nonetheless, the body of evidence on IBD patients’ tolerability resulting from the SATISFACTION randomized-controlled trial appears robust and very promising for the development of novel bowel preparation strategies targeting patients living with IBD. Prospective studies in larger IBD populations are now needed to confirm these findings and further evaluate the impact of same-day regimens and non-absorbable sugar laxatives.

In conclusion, our findings suggest that same-day OM preparation may enhance satisfaction and adherence in IBD patients, representing a potentially safe and effective alternative to standard protocols. If confirmed in larger, prospective studies, same-day preparation with OM will improve compliance and satisfaction among IBD patients, helping to optimize the quality of colonoscopy and the adherence to surveillance protocols for colitis-associated neoplasia.

## Data Availability

The raw data supporting the conclusions of this article will be made available by the authors, without undue reservation.
